# Naturally Occurring Single Mutations in Ebola Virus Observably Impact Infectivity

**DOI:** 10.1128/JVI.01098-18

**Published:** 2018-12-10

**Authors:** Gary Wong, Shihua He, Anders Leung, Wenguang Cao, Yuhai Bi, Zirui Zhang, Wenjun Zhu, Liang Wang, Yuhui Zhao, Keding Cheng, Di Liu, Wenjun Liu, Darwyn Kobasa, George F. Gao, Xiangguo Qiu

**Affiliations:** a Special Pathogens Program, Public Health Agency of Canada, Winnipeg, Manitoba, Canada; b Shenzhen Key Laboratory of Pathogen and Immunity, Guangdong Key Laboratory for Diagnosis and Treatment of Emerging Infectious Diseases, Second Hospital Affiliated to Southern University of Science and Technology, Shenzhen Third People’s Hospital, Shenzhen, China; c Department of Medical Microbiology, University of Manitoba, Winnipeg, Manitoba, Canada; d CAS Key Laboratory of Pathogenic Microbiology and Immunology, Institute of Microbiology, Center for Influenza Research and Early Warning (CASCIRE), Chinese Academy of Sciences, Beijing, China; e Monoclonal Antibody Unit, National Microbiology Laboratory, Public Health Agency of Canada, Winnipeg, Manitoba, Canada; Loyola University Medical Center

**Keywords:** Ebola virus, ferrets, mice, mutations, pathogenicity

## Abstract

Sequencing of Ebola virus (EBOV) genomes during the 2014–2016 epidemic identified several naturally occurring, dominant mutations potentially impacting virulence or tropism. In this study, we characterized EBOV variants carrying one of the following substitutions: A82V in the glycoprotein (GP), R111C in the nucleoprotein (NP), or D759G in the RNA-dependent RNA polymerase (L). Compared with the wild-type (WT) EBOV C07 isolate, NP and L mutants conferred a replication advantage in monkey Vero E6, human A549, and insectivorous bat Tb1.Lu cells, while L mutants displayed a disadvantage in human Huh7 cells. The replication of the GP mutant was significantly delayed in Tb1.Lu cells and similar to that of the WT in other cells. The L mutant was less virulent, as evidenced by increased survival for mice and a significantly delayed time to death for ferrets, but increased lengths of the period of EBOV shedding may have contributed to the prolonged epidemic. Our results show that single substitutions can have observable impacts on EBOV pathogenicity and provide a framework for the study of other mutations.

**IMPORTANCE** During the Ebola virus (EBOV) disease outbreak in West Africa in 2014–2016, it was discovered that several mutations in the virus emerged and became prevalent in the human population. This suggests that these mutations may play a role impacting viral fitness. We investigated three of these previously identified mutations (in the glycoprotein [GP], nucleoprotein [NP], or RNA-dependent RNA polymerase [L]) in cell culture, as well as in mice and ferrets, by generating recombinant viruses (based on an early West African EBOV strain) each carrying one of these mutations. The NP and L mutations appear to decrease virulence, whereas the GP mutation slightly increases virulence but mainly impacts viral tropism. Our results show that these single mutations can impact EBOV virulence in animals and have implications for the rational design of efficacious antiviral therapies against these infections.

## Abstract (precis)

During the Ebola virus (EBOV) disease outbreak in West Africa in 2014–2016, it was discovered that several mutations in the virus emerged and became prevalent in the human population. This suggests that these mutations may play a role impacting viral fitness. We investigated three of these previously identified mutations (in the glycoprotein [GP], nucleoprotein [NP], or RNA-dependent RNA polymerase [L]) in cell culture, as well as in mice and ferrets, by generating recombinant viruses (based on an early West African EBOV strain) each carrying one of these mutations. The NP and L mutations appear to decrease virulence, whereas the GP mutation slightly increases virulence but mainly impacts viral tropism. Our results show that these single mutations can impact EBOV virulence in animals and have implications for the rational design of efficacious antiviral therapies against these infections.

## INTRODUCTION

Ebola virus (EBOV) infections result in severe hemorrhagic fever with a case fatality rate as high as 90% (1), with death typically occurring 5 to 7 days after the appearance of symptoms (2). Past outbreaks of EBOV disease were localized primarily to the remote rainforests of sub-Saharan Africa and had limited impact on global human health (3). Despite the devastating consequences for affected communities, filoviruses were regarded as a minor public health threat, even in Africa (4). However, the 2014–2016 epidemic centered in Sierra Leone, Guinea, and Liberia was unprecedented in that a record number of people were infected (28,616) and killed (11,310) due to uncontrolled transmission of the virus within the local population (5). Furthermore, EBOV cases were imported into other countries in Africa, Europe, and North America, through travel or repatriation of infected patients, and occasionally resulted in further transmission of the virus (6). EBOV still constitutes an epidemic threat, as evidenced by the current (since May 2018) outbreak in the Democratic Republic of the Congo, which has already reached the city of Mbandaka and may spread to the capital, Kinshasa, by boat via the Congo River (7).

Next-generation sequencing of clinical samples collected from EBOV patients during the 2014–2016 epidemic has shown that certain naturally occurring single nucleotide polymorphisms (SNPs) in the EBOV genome have arisen at various times and have rapidly increased to high frequency, becoming dominant within the human population. These SNPs, which result in nonsynonymous mutations most of the time and are suspected to contribute to virulence, are as follows: (i) A82V in the glycoprotein (GP), which possibly impacts virus-host interactions, since it is located in the NPC-1 receptor binding site on EBOV GP, and *in vitro* studies using EBOV GP-pseudotyped viruses have shown that this mutation results in increased tropism for human-origin cell lines but decreased tropism for bat-origin cell lines (8, 9) and an increase in infectivity (10, 11); (ii) R111C in the nucleoprotein (NP), possibly impacting viral transcription/replication; and (iii) D759G in the RNA-dependent RNA polymerase (L), possibly impacting viral replication (12, 13). In addition, a paired T3008C T3011C mutation in the noncoding region between the NP and VP35 genes is suspected to enhance the expression of both the upstream and the downstream genes, as determined by the EBOV minigenome assay (12). Additionally, a T544I polymorphism in GP has been shown to modulate entry into host cells (14), although this mutation did not become fixed during the 2014–2016 epidemic (10). *In vitro* and *in vivo* characterization of the changes associated with these single mutations is needed to elucidate a mechanistic definition and confirm the preliminary findings from these studies.

In our study, we investigated the impact of three single nonsynonymous mutations: A82V in the GP, R111C in the NP, and D759G in L. More than 97% of known EBOV strains circulating in 2014–2016 contained the GP, NP, and/or L mutation. These mutations were generated based on the sequence of EBOV/C07 (designated the wild type [WT]), a West African isolate reported early during the 2014–2016 EBOV epidemic and now commonly used as a prototype West African EBOV for studies. To precisely identify the biological functions associated with the dominant mutations, cell lines with different host origins, including monkey (Vero E6), human (Huh7 and A549), and insectivorous bat (Tb1.Lu) cells, were used for *in vitro* studies, and two animal models, including type I interferon receptor-deficient (*Ifnar1^−/−^*) mice and ferrets, were used for *in vivo* studies. The results showed that these single mutations displayed observable differences in replication and virulence, suggesting an advantageous evolution of the virus for better replication and host adaptation.

## RESULTS

### Measuring viral growth kinetics by genome detection via qRT-PCR.

**FIG 1:**
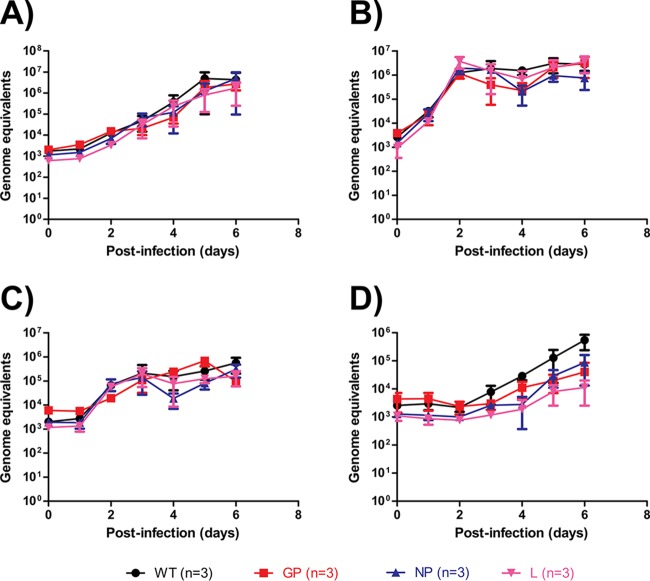
*In vitro* characterization of EBOV mutants via genome detection by qRT-PCR. Vero E6 (A), Huh7 (B), A549 (C), and Tb1.Lu (D) cells were infected with the various EBOV mutants at an MOI of 0.1. Samples were taken daily, and the RNA was extracted and quantified by qRT-PCR. RNA quantities are expressed as genome equivalents.

We next evaluated the growth kinetics of the mutants by genome detection via reverse transcription-quantitative PCR (qRT-PCR) in the Vero E6 (monkey kidney), Huh7 (human hepatocyte), A549 (human lung), and Tb1.Lu (insectivorous bat lung) cell lines. Cells were infected at a multiplicity of infection (MOI) of 0.1 and were monitored from day 0 (infection) to day 6. A “background” level of 10^3^ genome equivalents (GEQ) of EBOV, which corresponded to input virus, was detected at day 0. Titers in Vero E6 cells grew steadily to a peak of ∼10^6^ GEQ by day 5, and the growth curves were not statistically significant across the WT, GP mutant, NP mutant, and L mutant viruses (Fig. 1A). Titers in Huh7 cells grew rapidly to a peak of ∼10^6^ GEQ by day 2, and the growth curves were not statistically significant across the four viruses (Fig. 1B). Titers in A549 cells grew slowly to a peak of ∼10^5^ GEQ by day 2, and the growth curves were not statistically significant across the four viruses (Fig. 1C). Titers in Tb1.Lu cells grew slowly, with the GP, NP, and L mutants reaching levels slightly over ∼10^4^ GEQ by day 6, whereas the WT reached ∼10^5^ GEQ by the same day, and the growth curves were not statistically significant across the four viruses (Fig. 1D). Taken together, the data did not provide any obvious indications that the replication abilities of EBOV were substantially impacted by these mutations when evaluated by genome detection through qRT-PCR.

### Measuring viral growth kinetics via eGFP reporter gene detection.

**FIG 2:**
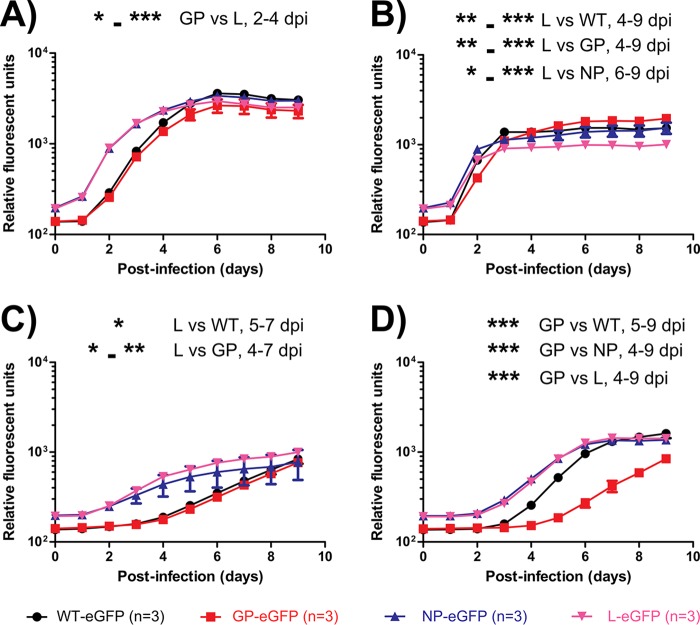
*In vitro* characterization of EBOV mutants as measured by eGFP reporter gene detection. Vero E6 (A), Huh7 (B), A549 (C), and Tb1.Lu (D) cells were infected with the various EBOV mutants expressing eGFP at an MOI of 0.1. Fluorescence readings were taken daily, and values are expressed as relative fluorescence units. The statistical significance of differences between groups, and the days in which values differed significantly, are indicated on the graphs (*, *P* < 0.05; **, *P* < 0.01; ***, *P* < 0.001).

We then evaluated the growth kinetics of the WT-eGFP, GP-eGFP, NP-eGFP, and L-eGFP viruses via detection of the enhanced green fluorescent protein (eGFP) reporter gene in the cell lines listed above. We infected cells with eGFP-expressing viruses as a surrogate for live virus titration, because this method is more robust and high-throughput for quantifying viral growth kinetics. Cells were infected at an MOI of 0.1 and were monitored from day 0 (infection) to day 9. A “background” level of 150 to 200 relative fluorescent units (RFU), corresponding to input virus, was observed on day 0. Titers in Vero E6 cells grew to a peak of ∼2 × 10^3^ RFU by day 9, although the NP-eGFP and L-eGFP viruses appear to have reached this peak faster than the GP-eGFP and WT-eGFP viruses. Supporting this trend are the significantly lower RFU values for the GP-eGFP virus than for the L-eGFP virus at days 2 to 4 after infection (Fig. 2A). Titers in Huh7 cells grew to a peak of ∼10^3^ RFU around day 3, and all four viruses reached plateaus at similar times. Cells infected with the L-eGFP virus reached a lower plateau than those infected with the WT, GP mutant, or NP mutant virus, and the difference was significant between day 4 or 6 and day 9 (Fig. 2B). Titers in A549 cells grew to a peak of ∼10^3^ RFU by day 9, and the NP-eGFP and L-eGFP viruses appear to have reached this peak faster than the GP-eGFP and WT-eGFP viruses. Significantly lower RFU values were observed for the GP-eGFP and WT-eGFP viruses than for the L-eGFP virus between day 4 or 5 and day 7 (Fig. 2C). Titers in Tb1.Lu cells grew to a peak of ∼10^3^ RFU by day 7, except for the GP-eGFP virus, which grew much more slowly. Significantly lower RFU values were observed for the GP-eGFP virus between day 4 or 5 and day 9 than for the other three viruses (Fig. 2D). Taken together, the data suggest that the L and NP mutations confer a replication advantage over the WT and the GP mutant in Vero E6 and A549 cells, the L mutation confers a replication disadvantage in Huh7 cells, and the GP mutation results in a replication disadvantage in Tb1.Lu cells.

### Characterization of mutants in *Ifnar1^−/−^* mice.

**FIG 3:**
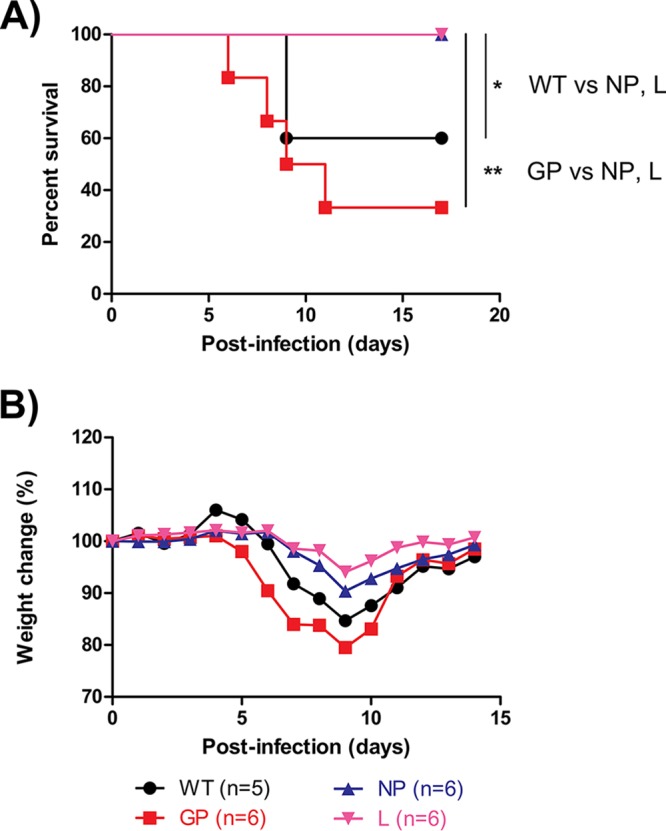
*In vivo* characterization of EBOV mutants in *Ifnar1^−/−^* mice. The *Ifnar1^−/−^* mice were inoculated intraperitoneally with 0.1 PFU of the various EBOV mutants and were monitored daily thereafter for survival (A) and average weight loss (B). The statistical significance of differences between groups is indicated only when *P* values were <0.05 (*, *P* < 0.05; **, *P* < 0.01).

In order to determine whether these mutations had any observable effects, they were evaluated in *Ifnar1^−/−^* mice. The 50% lethal dose (LD_50_) of the WT was determined to be 0.078 PFU from a previous study (15). Groups of 5 to 6 mice were given 0.1 PFU of the WT or the GP, NP, or L mutant via the intraperitoneal (i.p.) route and were monitored for survival and weight loss. WT- and GP mutant-infected mice had 60% survival (3/5 mice) and 33% survival (2/6 mice), respectively (Fig. 3A), with mice dying between 6 and 11 days after infection. Surprisingly, infections with the NP or L mutant were less lethal to these mice, with 100% survival (6/6 mice) in both groups, and the differences were significant (Fig. 3A). The weight loss data support the survival observations: mice infected with the L mutant, the NP mutant, the WT, or the GP mutant lost at most 6%, 10%, 16%, or 20% of body weight on average, respectively (Fig. 3B). Taken together, the data suggest that the L and NP mutants were less pathogenic than the GP mutant and the WT in mice.

### Characterization of mutants in domestic ferrets.

**FIG 4:**
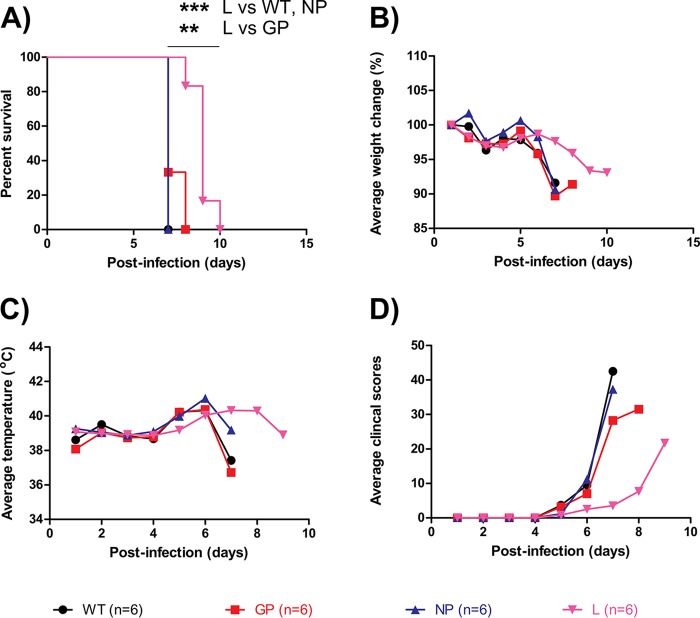
*In vivo* characterization of EBOV mutants in domestic ferrets. Domestic ferrets were inoculated intramuscularly with a target 0.02 PFU of the various EBOV mutants and were monitored daily thereafter for survival (A), weight loss (B), body temperature (C), and clinical score (D). The statistical significance of differences between groups is indicated only where *P* values were <0.05 (**, *P* < 0.01; ***, *P* < 0.001).

These mutations were further evaluated in domestic ferrets. The LD_50_ of the WT was determined to be 0.015 PFU from a previous study (15). Groups of 6 ferrets were given a target of 0.02 PFU of the WT or the GP, NP, or L mutant (back-titrated values were comparable at 0.04, 0.03, 0.06, and 0.09 PFU, respectively) via the intramuscular (i.m.) route. Ferrets were shown to be susceptible to infection, with all WT- and NP mutant-infected animals succumbing at day 7 (average time to death, 7 ± 0 days), all GP mutant-infected animals dying by day 8 (average time to death, 7.3 ± 0.5 days), and all L mutant-infected animals dying between days 8 and 10 (average time to death, 9.0 ± 0.6 days). The difference between L mutant-infected animals and the other groups was significant (Fig. 4A). WT-, GP mutant-, and NP mutant-infected animals lost an average ∼10% of body weight by day 7 (the day of death), whereas weight loss was delayed and milder in L mutant-infected animals (Fig. 4B). WT-, GP mutant-, and NP mutant-infected animals all developed fever (above 40°C) by day 6, whereas the development of fever was not as noticeable in L mutant-infected animals (Fig. 4C). Additionally, WT-, GP mutant-, and NP mutant-infected animals had considerably higher clinical scores by day 7, necessitating euthanasia, whereas L mutant-infected animals were relatively symptom-free on the same day (Fig. 4D). Taken together, these observations show that the pathogenesis in L mutant-infected ferrets is slower than that with the other three viruses.

**FIG 5:**
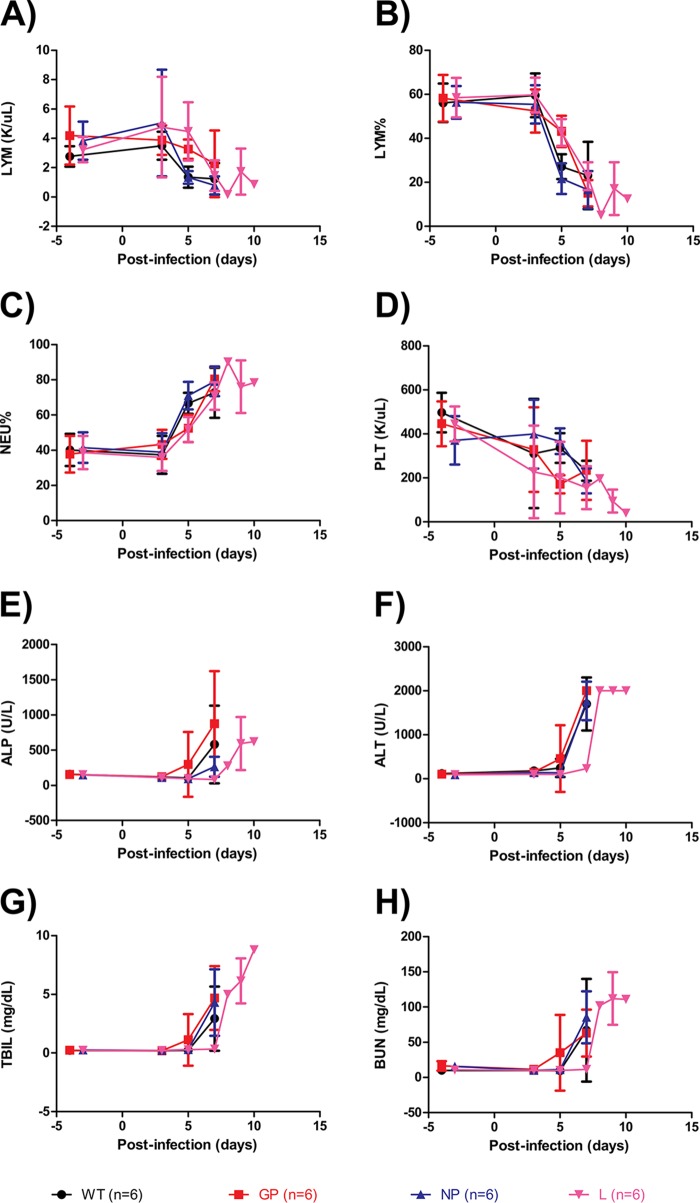
Hematological and serum biochemical values after infection of ferrets with various EBOV mutants. The parameters shown are the lymphocyte (LYM) count (A), lymphocyte percentage (LYM%) (B), neutrophil percentage (NEU%) (C), platelet (PLT) count (D), alkaline phosphatase (ALP) activity (E), alanine aminotransferase (ALT) activity (F), total-bilirubin (TBIL) concentration (G), and blood urea nitrogen (BUN) concentration (H).

Hematological and biochemical parameters were also measured on selected days before and after challenge. The EBOV-infected ferrets followed a typical filoviral disease course, with marked decreases in lymphocyte (LYM) counts, the percentage of lymphocytes (LYM%), and platelet (PLT) counts, but a marked increase in the percentage of neutrophils (NEU%), alkaline phosphatase (ALP) activity, alanine aminotransferase (ALT) activity, total-bilirubin (TBIL) concentrations, and blood urea nitrogen (BUN) concentrations over time. While WT-, GP mutant-, and NP mutant-infected animals all showed similar perturbations to these hallmark parameters at similar times, L mutant-infected animals showed a 2-day delay in some parameters, namely, ALP and ALT activities and TBIL and BUN concentrations (Fig. 5A to H). The severity of filoviral disease, according to these parameters, was similar for all four groups at the time of death.

**FIG 6:**
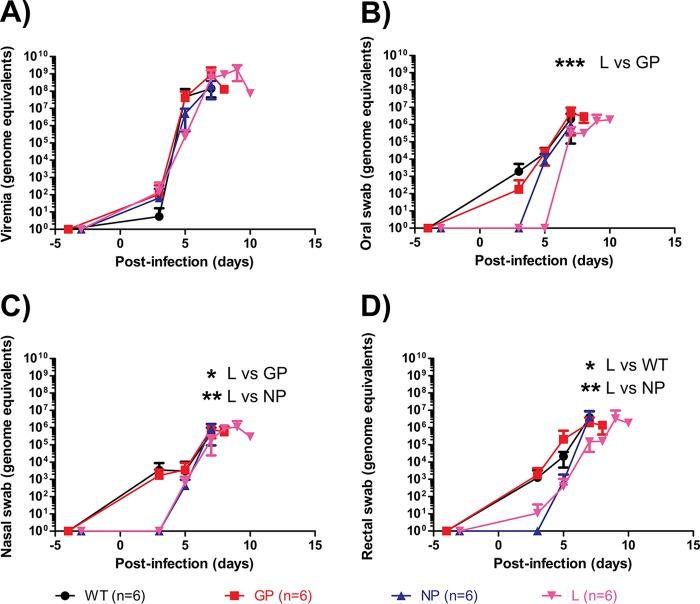
Viremia and shedding in ferrets after infection with various EBOV mutants. The levels of viremia (A) and virus shedding via the oral (B), nasal (C), and rectal (D) routes were quantified by qRT-PCR and are expressed as genome equivalents. The statistical significance of differences between groups, and the days on which values differed significantly, are indicated on the graphs only when *P* values were <0.05 (*, *P* < 0.05; **, *P* < 0.01; ***, *P* < 0.001).

Viremia and virus shedding from the oral, nasal, and rectal routes were quantified at selected times after infection. Peak viremia up to ∼10^8^ to ∼10^9^ GEQ can be observed in animals at the terminal stages of disease. Notably, the level of viremia at day 5 was 100- to 1,000-fold lower for L mutant-infected animals than for WT-, GP mutant-, or NP mutant-infected animals (Fig. 6A). Oral shedding was detected by day 3 for WT- and GP mutant-infected animals, by day 5 for NP mutant-infected animals, and by day 7 for L mutant-infected animals, reaching a peak of ∼10^6^ GEQ for all groups by the time of death (Fig. 6B). Nasal shedding was detected by day 3 for WT- and GP mutant-infected animals and by day 5 for NP mutant- and L mutant-infected animals, reaching a peak of ∼10^6^ GEQ for all groups by the time of death (Fig. 6C). Rectal shedding was detected by day 3 for WT-, GP mutant-, and L mutant-infected animals and by day 5 for NP mutant-infected animals, reaching a peak of ∼10^6^ GEQ for all groups by the time of death (Fig. 6D). Blood samples (at 7 days postinfection [dpi] and terminal bleeds) from all animals were processed by Sanger sequencing, and no reversions to wild type were found for the EBOV mutants.

**FIG 7:**
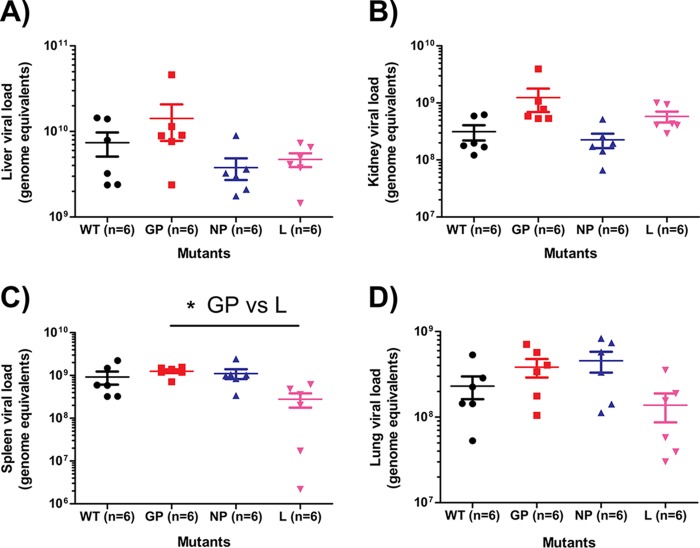
Biodistribution of various EBOV mutants in the major organs of ferrets at the point of euthanasia or death. The levels of EBOV in the liver (A), kidney (B), spleen (C), and lung (D) were quantified by qRT-PCR and are expressed as genome equivalents. The statistical significance of differences between groups is indicated only when *P* values were <0.05 (*).

To assess the biodistribution of the virus in ferrets at the time of death, the liver, kidney, spleen, and lung were harvested and the viral RNA quantified. Virus was found to be most abundant in the liver, at ∼10^10^ GEQ, followed by the spleen at ∼10^9^ GEQ and then the kidney and lung at ∼10^8^ to ∼10^9^ GEQ. The titers for the different virus groups were comparable, and any differences were generally not statistically significant (Fig. 7A to D). Taking the data together, after infection with EBOV at comparable titers, L mutant-infected ferrets had a significantly delayed time to death (48 h) relative to that for WT-, GP mutant-, and NP mutant-infected animals. These observations were supported by all hematological, biochemical, and virologic parameters tested.

## DISCUSSION

The 2014–2016 epidemic of EBOV disease was the largest on record, and it is likely that a combination of several factors was responsible, including substandard levels of medical care, high population mobility, and traditional beliefs and behavioral practices (16). Additionally, the possibility that EBOV variants from West Africa are more pathogenic than their Central African counterparts has been a topic of intense debate (17, 18). The prolonged nature of the EBOV disease epidemic in West Africa means that the virus has gone through many “passages” in humans and, as a result, may have evolved and/or adapted to its human hosts over time. Several nonsynonymous mutations rapidly became fixed in the population after their emergence. Studies with a pseudotyped virus carrying the GP mutation only, as well as *in vitro* studies with a live virus carrying all three of the GP, NP, and L mutations, appear to support enhanced virulence. However, there are several remaining issues to solve. First, a mechanistic definition of the impact of each dominant mutation is lacking. Second, it is not known whether these changes translate to observable differences *in vivo*.

To address these issues, we took a methodical approach in studying each mutation in isolation, using *in vitro* and *in vivo* models. We chose the Vero E6, A549, Huh7, and Tb1.Lu cell lines because previous publications had shown that they are susceptible to *in vitro* infection with EBOV (13, 19, 20). It is possible that the inclusion of primary cells from a known EBOV target cell type or a fruit bat cell line (such as R06E [21]) instead of the insectivorous bat cell line Tb1.Lu may yield even more discriminatory findings. Our *in vitro* data suggest that the L and NP mutants have a replication advantage in Vero E6 and A549 cells over the WT and the GP mutant, whereas the L mutant also has a replication disadvantage in Huh7 cells compared to the WT and the GP and NP mutants. While it is possible that combinations of mutations do not necessarily operate synergistically, these results are consistent with previous *in vitro* data, in which infection of the Vero E6 and Huh7 cell lines by a live EBOV mutant simultaneously carrying the GP, NP, and L mutations would be expected to result in enhanced replication overall (13). *In vitro* and *in vivo* characterization of a natural isolate or a recombinant virus carrying a combination of these three mutations should be undertaken in the future to determine whether the combination of these mutations has synergistic or antagonistic effects. Additionally, whether certain mutations can amplify observed phenotypes is an interesting topic of investigation. Interestingly, an attenuating phenotype was observed for the L mutation. A literature search showed that this mutation is in the RNA-dependent RNA polymerase catalytic domain, located 15 amino acids past the catalytic center (GDNQ motif) that is conserved among negative-stranded viruses. Abrogation of the catalytic center was found to completely inhibit viral genome replication and transcription in a previous study (22). Although the exact site of the L mutation was not conserved among negative-stranded viruses, it is possible that this amino acid residue contributes to the structural stability of the catalytic domain, a possibility that should be investigated in future studies.

For *in vivo* studies, *Ifnar1^−/−^* mice were used first, since mice with this phenotype have been shown to be highly susceptible to infection by wild-type filoviruses, with death occurring within 5 to 7 days and a disease course similar to those of humans and nonhuman primates (NHPs), even after challenge at low doses of EBOV (15). Due to their lower costs and less stringent requirements for housing and handling, these animals may be a more convenient model with which to screen individual mutations. Additionally, we challenged all animals with a low dose of EBOV (approximating 1× LD_50_, or ∼0.08 PFU in *Ifnar1^−/−^* mice and 0.02 PFU in ferrets) rather than the high dose of EBOV (1,000 PFU) typically employed for therapeutic or vaccine studies. This is due to the considerations that (i) high doses of EBOV are typically used to ensure uniform lethality of animals at approximately 7 days after infection and (ii) doses as low as 1 PFU EBOV can be lethal to NHPs, and significant differences in survival have been observed between different infection groups (23). Low doses may be more conducive to detecting small variations in phenotype, which may not be observed in *Ifnar1^−/−^* mice given at least 100 to 100,000 focus-forming units (FFU) of virus, or in NHPs given 1,000 FFU (24). Our results strongly indicate that the L mutation decreases the virulence of EBOV *in vivo*, as shown with mice and ferrets. The increased length of virus shedding seen in these ferrets may contribute to increased virus transmission, thereby providing an explanation for the increased numbers of cases observed during the 2014–2016 epidemic. The NP mutation decreases *in vivo* virulence, as shown by the mouse studies. The GP mutation appears to increase *in vivo* virulence, as shown by the mouse studies, although the difference is not statistically significant.

It will be of interest to explore in more depth why the various mutants behaved differently *in vitro* than *in vivo*. A possible factor is that neither cell culture, *Ifnar1^−/−^* mice, nor domestic ferrets can fully and accurately recapitulate human EBOV disease, thus leading to conflicting outcomes. Findings should ideally be confirmed by more than one animal model, preferably including the “gold standard,” NHPs, but it is likely that the heterogeneity in human populations cannot be represented in a small set of macaques of similar ages, weights, etc. It is also possible that the complex environment of novel mutations in relation to the variable immune systems of different individuals could, for now, only be grossly modelled. In this regard, this study provides data associated with different experimental systems that, when used in conjunction with data from other systems, could lead to the identification of better conditions for detecting phenotypes and associating them with genotypes (and vice versa). This study provides a solid platform for screening any virulence changes associated with other dominant mutants, and the findings identify potential “weak spots” in EBOV, which will be important for the design of effective antivirals as well as for surveillance studies.

## MATERIALS AND METHODS

### Generation of EBOV mutants.

Mutants were generated based on the Makona EBOV/C07 isolate (GenBank accession no. KJ660347.2) isolated early in the 2014–2016 epidemic, using a modified in-house reverse genetics system (15). We generated eight different viruses for this study: EBOV/C07 (WT), EBOV/C07-A82V/GP (GP mutant), EBOV/C07-R111C/NP (NP mutant), and EBOV/C07-D759G/L (L mutant), as well as their eGFP-expressing counterparts EBOV/C07-eGFP (WT-eGFP), EBOV/C07-A82V/GP-eGFP (GP-eGFP), EBOV/C07-R111C/NP-eGFP (NP-eGFP), and EBOV/C07-D759G/L-eGFP (L-eGFP). After rescue, virus stocks were prepared and the viral genome confirmed by next-generation sequencing, as described previously (15). The viruses without eGFP were used for *in vitro* characterization studies by genome detection via qRT-PCR as well as for all *in vivo* characterization experiments, whereas the eGFP-expressing viruses were used for *in vitro* characterization studies via eGFP reporter gene measurement.

### Measuring viral growth kinetics by genome detection via qRT-PCR.

Cell lines originating from humans (Huh7 and A549), monkeys (Vero E6), or insectivorous bats (Tb1.Lu) were grown and maintained at 37°C in Dulbecco’s modified Eagle medium (DMEM) supplemented with 10% fetal bovine serum (FBS), 2 mmol/liter of l-glutamine, 100 U/ml of penicillin, and 100 μg/ml of streptomycin. Cells were seeded in 6-well plates and were approximately 95% confluent at the time of infection. The cells were then infected in triplicate with the WT or the GP, N, or L mutant at a multiplicity of infection (MOI) of 0.1 in a volume of 0.5 ml and were incubated at 37°C for 1 h, and the inoculum was replaced with 3 ml of DMEM supplemented with 2% FBS. Supernatants (250 µl) were harvested daily from 0 to 6 days after infection and were replaced with a corresponding amount of DMEM supplemented with 2% FBS. Genomic material was detected using the LightCycler 480 RNA Master Hydrolysis Probes (Roche) kit, targeting the RNA polymerase gene (nucleotides 16472 to 16538; GenBank accession no. AF086833). PCR conditions were as follows: 63°C for 3 min, 95°C for 30 s, and cycling of 95°C for 15 s and 60°C for 30 s for 45 cycles on the ABI StepOnePlus thermocycler. The primers used (with sequences in parentheses) were as follows: EBOVLF2 (CAGCCAGCAATTTCTTCCAT), EBOVLR2 (TTTCGGTTGCTGTTTCTGTG), and EBOVLP2FAM (6-carboxyfluorescein [FAM]-ATCATTGGCGTACTGGAGGAGCAG-black hole quencher 1 [BHQ1]). The data are presented in genome equivalents (GEQ).

### Measuring viral growth kinetics via eGFP reporter gene detection.

Huh7, A549, Vero E6, and Tb1.Lu cells were seeded in 96-well plates and were approximately 95% confluent at the time of infection. The cells were infected in triplicate with the WT-eGFP, GP-eGFP, N-eGFP, or L-eGFP virus at an MOI of 0.1 in a volume of 250 µl and were incubated at 37°C for 1 h, and the inoculum was replaced with 100 µl of DMEM supplemented with 2% FBS. The expression of eGFP was then measured daily using the Synergy HT microplate reader (BioTek), and the data are presented in relative fluorescent units (RFU).

### Characterization of mutants in mice.

Immunocompromised (*Ifnar^−/−^*) male and female mice on the C57BL/6 background, ranging in age from 4 to 8 weeks, were obtained commercially (Jackson Laboratory). The mice were infected intraperitoneally (i.p.) with 0.1 PFU of the WT or the GP, N, or L mutant in a volume of 0.2 ml. Survival and weight loss were monitored daily for 17 days. Animals losing >20% of their initial body weight as determined on day 0 were euthanized according to animal ethics guidelines.

### Characterization of mutants in ferrets.

Immunocompetent domestic ferrets (Mustela putorius furo) were challenged intramuscularly (i.m.) with a predetermined low dose (target, 1× LD_50_, or 0.03 PFU) (15) of the WT virus or the GP, N, or L mutant in a volume of 1 ml. Survival, body temperature, and weight loss, as well as clinical symptoms such as decreased appetite and behavioral changes, were observed daily. Additionally, hematological, biochemical, and virological (viremia, shedding, and biodistribution) parameters were monitored on exam days (days –5 or –4, 3, 5, and 7 and the day of euthanasia or death). Animals with a clinical score of >20 were euthanized according to animal ethics guidelines.

### Ethics statement.

All experiments involving live, infectious EBOV were performed at the biosafety level 4 (BSL-4) laboratory located at the National Microbiology Laboratory, Public Health Agency of Canada, Winnipeg. All animal experiments were performed according to guidelines set forth by the Animal Care Committee (ACC) in accordance with the Canadian Council on Animal Care (CCAC).

### Sample processing.

Complete blood counts and blood biochemistry analyses were performed on whole-blood and serum specimens, respectively, using the VetScan HM5 or VetScan VS2 system (Abaxis Veterinary Diagnostics) according to manufacturer instructions. Levels of viremia and shedding were determined by qRT-PCR. Total RNA was extracted from whole-blood samples with the QIAamp viral RNA minikit (Qiagen) and was quantified as described above. Major organs (liver, kidney, spleen, and lung) were harvested from all euthanized ferrets, and total RNA was extracted using the RNeasy minikit (Qiagen) and was quantified as described above.

### Statistical analysis.

All graphs were generated using GraphPad Prism, version 5. Viral growth kinetics, as determined by genome detection via qRT-PCR or by eGFP reporter gene detection, as well as viremia and shedding in ferrets, were analyzed using two-way analysis of variance (ANOVA) with the Bonferroni posttest. The log-rank (Mantel Cox) test was used to compare survival curves. The biodistribution of virus in euthanized ferrets was analyzed using one-way ANOVA with the Bonferroni posttest. The statistical significance of differences between groups, and the days on which values differed significantly, are shown on the graphs only when *P* values were <0.05.
